# Transcriptomes of the tumor-adjacent normal tissues are more informative than tumors in predicting recurrence in colorectal cancer patients

**DOI:** 10.1186/s12967-023-04053-2

**Published:** 2023-03-21

**Authors:** Jinho Kim, Hyunjung Kim, Min-Seok Lee, Heetak Lee, Yeon Jeong Kim, Woo Yong Lee, Seong Hyeon Yun, Hee Cheol Kim, Hye Kyung Hong, Sridhar Hannenhalli, Yong Beom Cho, Donghyun Park, Sun Shim Choi

**Affiliations:** 1 Precision Medicine Center, Future Innovation Research Division, Seoul National University Bundang Hospital, Seongnam, 13620 Korea; 2 Division of Biomedical Convergence, College of Biomedical Science, Institute of Bioscience & Biotechnology, Kangwon National University, Chuncheon, 24341 Korea; 3 Center for Genome Engineering, Institute for Basic Science, 55, Expo-ro, Yuseng-gu, Daejeon, 34126 Korea; 4 Samsung Genome Institute, Samsung Medical Center, Seoul, 06351 Korea; 5 Department of Surgery, Samsung Medical Center, Sungkyunkwan University School of Medicine, Seoul, 06351 Korea; 6 Institute for Future Medicine, Samsung Medical Center, Seoul, 06351 Korea; 7 Cancer Data Science Lab, Center for Cancer Research, National Cancer Institute, Bethesda, 20814 MD USA; 8 Department of Health Sciences and Technology, SAIHST, Sungkyunkwan University, Seoul, 06351 Korea; 9 Geninus Inc., Seoul, 05836 Korea

**Keywords:** Colorectal cancer, Normal tissues adjacent to tumors, Recurrence, Elastic net-based machine learning, Tumor-infiltrating immune cells

## Abstract

**Background:**

Previous investigations of transcriptomic signatures of cancer patient survival and post-therapy relapse have focused on tumor tissue. In contrast, here we show that in colorectal cancer (CRC) transcriptomes derived from normal tissues adjacent to tumors (NATs) are better predictors of relapse.

**Results:**

Using the transcriptomes of paired tumor and NAT specimens from 80 Korean CRC patients retrospectively determined to be in recurrence or nonrecurrence states, we found that, when comparing recurrent with nonrecurrent samples, NATs exhibit a greater number of differentially expressed genes (DEGs) than tumors. Training two prognostic elastic net-based machine learning models—NAT-based and tumor-based in our Samsung Medical Center (SMC) cohort, we found that NAT-based model performed better in predicting the survival when the model was applied to the tumor-derived transcriptomes of an independent cohort of 450 COAD patients in TCGA. Furthermore, compositions of tumor-infiltrating immune cells in NATs were found to have better prognostic capability than in tumors. We also confirmed through Cox regression analysis that in both SMC-CRC as well as in TCGA-COAD cohorts, a greater proportion of genes exhibited significant hazard ratio when NAT-derived transcriptome was used compared to when tumor-derived transcriptome was used.

**Conclusions:**

Taken together, our results strongly suggest that NAT-derived transcriptomes and immune cell composition of CRC are better predictors of patient survival and tumor recurrence than the primary tumor.

**Supplementary Information:**

The online version contains supplementary material available at 10.1186/s12967-023-04053-2.

## Background

Histologically normal tissues adjacent to the tumors (NATs) have long been considered equivalent to a healthy normal [1]. It is thus a common practice to use NAT as a control for tumor in omics studies [2–4], although the difficulty in acquiring healthy tissue is a strong reason underlying this practice. However, recent studies have shown that NAT has molecular and cell compositional properties distinct from healthy normal tissues, positioned in an intermediate state between healthy normal and tumor tissues, which can differentiate poor or good prognosis of cancers [5, 6].

Colorectal cancer (CRC) is one of the most commonly diagnosed cancers worldwide [7]. As a result of increased cancer screening at the population level, early CRC can be treated with surgical removal of the tumor combined with chemotherapy [8]. However, approximately 30–50% of CRC patients are predicted to eventually experience recurrence and metastasis after treatment, with a 5-year survival rate of less than 60% [9–12]. The mechanism driving recurrence after surgical resection of CRC remains unclear. In terms of biomarkers to predict patient prognosis, TNM staging method, a method integrating tumor (T), lymph node (N) and metastases (M), is commonly used to classify cancers pathologically by their localization and histology [13–15]. However, TNM staging often fails to predict the prognosis of patients after treatments. Mutational subtyping of CRC has thus been developed for targeted therapy such as anti-EGF receptor antibody called cetuximab, with limited success [16, 17].

To overcome these challenges, numerous studies have tried to classify tumors based on various molecular markers of CRC including microsatellite instability (MSI) [18, 19], CpG island methylator phenotype (CIMP) [20, 21], chromosomal instability (CIN) [22, 23], and *BRAF* and *KRAS* mutations [24, 25]. Compositional changes in stromal and mesenchymal cell subpopulations and patterns of tumor infiltrating B cells, T cells, and myeloid cells are another area of research looking for the CRC prognosis markers [26, 27]. Furthermore, several recent studies have shown that consensus molecular subtypes (CMS) (including CMS1 through CMS4), a system that was developed by integrating gene expressions of cell-type specific marker genes, key mutations events, and cell subpopulations in tumor microenvironment, is associated with therapy response, patient prognosis, and tumor recurrence [28–30]. However, CMS subtyping is also uncertain for ~ 63% of CRC samples [31–35].

It is noteworthy that a majority of these studies regarding the prognostic classifications described above has been performed with data derived from tumor tissues and the tumor microenvironments. However, previous studies have shown that NATs are quite distinct from healthy normal in their molecular makeup and various degrees of transcriptional similarities to the tumor in different types of cancers [1–6], and furthermore, NATs may represent the tissue microenvironmental changes facilitating tumor growth [36] and therefore may be informative with regards to patient prognosis and drug response and recurrence. For instance, according to Graham et al. [37], gene expressions in NATs can identify estrogen receptor (ER)-positive and ER-negative breast cancers. In addition, Pan et al. [5] have reported Hippo-related genes expressed in NATs to harbor prognostic property in hepatocellular carcinomas.

Here we investigate specifically in CRC relative advantage of NAT transcriptome over the tumor transcriptome in clinical prognosis. For this purpose, using the CRC-derived NAT and tumor paired transcriptome data generated by Samsung Medical Center (SMC) in Korea, we build two classes of elastic net-based machine-learning models, i.e., NAT-based models and tumor tissue-based model, to predict CRC prognosis, and examine which of the two types of models predict better the recurrence states of CRC patients, i.e., recurrent (shorten to be RC) and nonrecurrent (shorten to be nonRC) states. We validated the models built with SMC-derived transcriptomes in independent transcriptome data of The Cancer Genome Atlas (TCGA)-colorectal adenocarcinoma (COAD) cohort. We believe that our study substantially contributes toward establishing NATs as a critical resource to understand oncogenesis, tumor aggressiveness, and therapy response.

## Results

### Summary characteristics of tissue specimens to produce total RNA-seq data

**Fig. 1:**
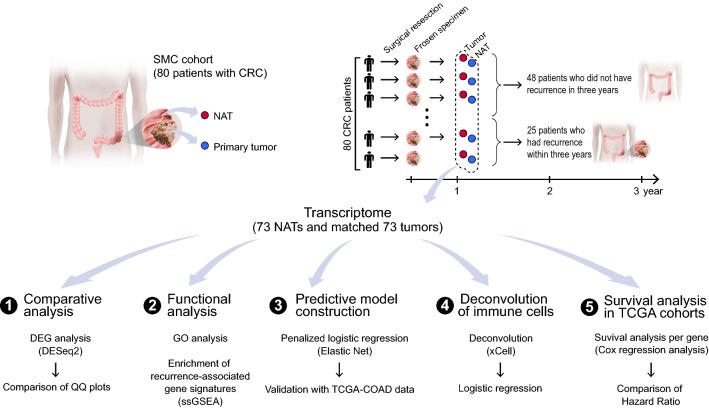
Study overview. RNA-seq data were produced from 160 surgical tumor and NAT samples from 80 Korean CRC patients. The total RNA-seq data, (i) tumor-derived transcriptomes and (ii) NAT-derived transcriptomes, were used to identify DEGs by comparing between RC and nonRC groups and functional analysis was done for the DEGs. The RNA-seq data of DEGs were used to construct recurrence prediction machine learning models. Subsequently, we investigated which machine learning prediction models constructed with NAT-based datasets or tumor-based datasets were superior for differentiating the recurrence states of patients with CRC. The two types of prediction models were then validated using the tumor-derived transcriptome data of 450 TCGA-COAD samples. Inferred immune cell composition were also used to compare which type of samples has more significant association with recurrence states with respect to infiltrated immune cell compositions. Finally, association of each gene with the survival of patient in different cancer types of TCGA cohorts was compared between NATs and tumor tissues

A total of 80 Korean patients with primary CRC determined with TNM stages 1–3 after excluding TNM stage 4 who did not have distant metastasis at the time of surgical resection were enrolled in this study. A total of 160 tissue specimens from the 80 patients, including primary tumor tissues and adjacent histologically normal tissues (i.e., NATs) derived from the same individuals were collected during the resection surgery, operated in SMC from the period, 2011 to 2013 (Table 1). The average size of the resected primary tumors was about 6.2 cm, and the NATs were biopsied at approximately 10 cm from the tumor resection boundary. Most samples (72/80; 90.0%) with an average age of about 63.86 were from male patients (Table 1). About 87.5% (70/80) of the samples were microsatellite stable (MSS) while only 12.5% (10/80) had high microsatellite instability (MSI-H) (Table 1); MSI was determined by PCR when two or more of the five repetitive sequences (BAT25, BAT26, D2S123, D5S346, D17S250) were unstable with indels, otherwise the sample was deemed MSS [38]. After collecting samples from surgical resection, patients’ progress was followed up for three years to examine whether recurrence occurred. As a result, a total of 73 patients including 25 patients with recurrence and 48 patients with nonrecurrence were used for further analyses (Fig. 1), after removing seven samples with ambiguity in the recurrence state; two different types of total RNA-seq data were generated from each of the 146 specimens, i.e., 73 tumor-derived transcriptomes and 73 site paired NAT-derived transcriptomes. Based on these two types of transcriptomes, we later developed machine learning models for predicting the recurrence of patients with CRC (Fig. 1).

**Table 1:** Patient characteristics

Variable	nonRC, N = 48^a^	RC, N = 25^a^	Unknown, N = 7^a^	*P*-value^b^
Sex				0.4
F	6 (12%)	1 (4.0%)	1 (14%)	
M	42 (88%)	24 (96%)	6 (86%)	
Age	62 (51, 70)	66 (51, 74)	75 (64, 77)	0.14
TNM stage				0.2
0	1 (2.1%)	0 (0%)	0 (0%)	
I	4 (8.3%)	2 (8.0%)	1 (14%)	
IIA	19 (40%)	5 (20%)	1 (14%)	
IIB	1 (2.1%)	0 (0%)	0 (0%)	
IIC	1 (2.1%)	0 (0%)	0 (0%)	
IIIA	3 (6.2%)	0 (0%)	0 (0%)	
IIIB	17 (35%)	11 (44%)	4 (57%)	
IIIC	2 (4.2%)	7 (28%)	1 (14%)	
MSI				0.045
MSI-H	9 (19%)	0 (0%)	1 (14%)	
MSS	39 (81%)	25 (100%)	6 (86%)	

^a^n(%); Median (IQR)

^b^For dichotomous variables (ex. Sex, MSI), Fisher’s exact test was used, while for variables with more than two groups such as Age, TNM stage, Kruskal–Wallis rank sum test was employed

### Relative to tumors, NATs exhibit smaller magnitudes but significant differences in expression between RC and nonRC states

**Fig. 2:**
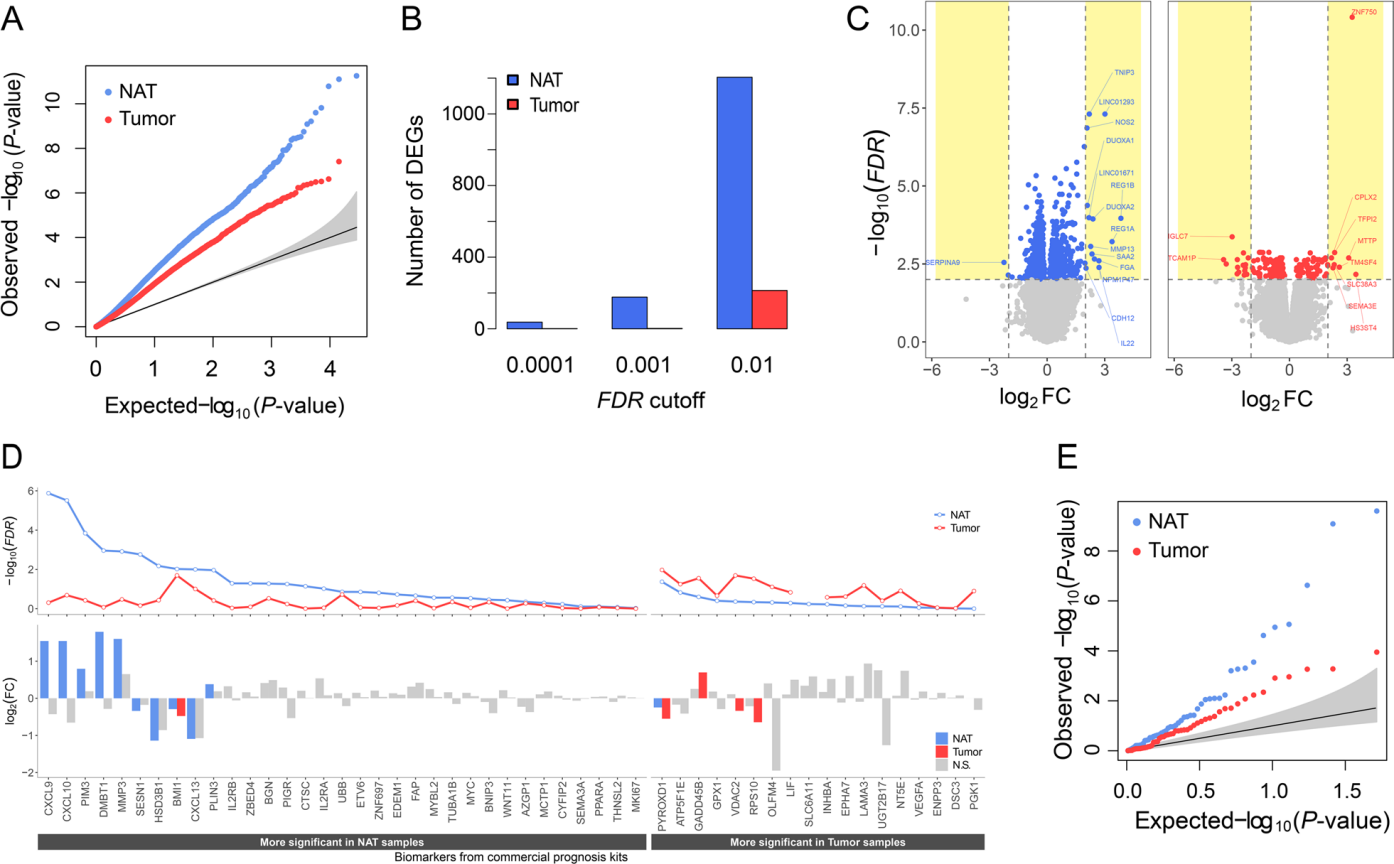
Comparison of the usefulness of NATs and tumor tissues for discovering prognostic gene markers. **A**–**C** Two types of DEGs, NAT-DEGs and tumor-DEGs, were identified by comparing gene expression levels between RC and nonRC samples using the NAT-derived transcriptomes and tumor-derived transcriptomes. **A** QQ plots comparing *P*-values of NAT-DEGs and tumor-DEGs. **B** Bar graphs showing the number of NAT-DEGs and tumor-DEGs obtained with three FDR cutoffs (0.0001, 0.001, and 0.01). **C** Volcano plots generated by selecting NAT-DEGs (left) and tumor-DEGs (right) based on the thresholds of − log_10_(*FDR*) > 2. The names of selected DEGs identified with these thresholds are shown. Yellow shaded areas represent − log_10_(*FDR*) > 2 and abs(log_2_FC) > 2. **D** Comparison of the − log_10_(*FDR*) (upper panel) and log_2_FC (lower panel) of the changes in gene expression between RC and nonRC states of 51 previously identified prognostic marker genes in NATs and tumors. Genes with lower *FDR* values in NAT than in tumor are on the left panel, and vice versa on the right panel. Note that *P-*values rather than *FDR* values were assigned to *SLC6A11*, in either NAT-derived transcriptome or tumor-derived transcriptome, because due to too low mean counts for this gene resulting in ‘NA’ by DESeq2. The grey bars on the lower panels represent the genes with no statistical difference between NAT and tumors (*FDR* > 0.05). N.S., not significant. **E** QQ plot of P-values estimated from (**D**) in NATs and tumors

We first investigated how the two types of data, tumor-derived transcriptomes and NAT-derived transcriptomes, transcriptionally differentiate RC from nonRC states with respect to the numbers and the statistical significance of DEGs. For this purpose, we derived two types of DEGs; (i) tumor-DEGs, i.e., DEGs estimated using the tumor-derived transcriptome by comparing the expression levels of genes between RC and nonRC samples, and (ii) NAT-DEGs, i.e., DEGs estimated using the NAT-derived transcriptome by comparing the expression levels of genes between RC and nonRC samples. We found that, in both types of transcriptomes, the *P-*values of DEGs deviated substantially from random expectations in the QQ plots (Fig. 2A), however, the magnitude of *P*-value deviations for the NAT-DEGs was far greater than that for the tumor-DEGs (Fig. 2A). Consistently, the numbers of NAT-DEGs were significantly larger than those of tumor-DEGs at various false discovery rate (*FDR*) thresholds from *FDR* < 0.01 to *FDR* < 0.0001 (Fig. 2B). Notably, at *FDR* < 0.01, almost six times more NAT-DEGs than tumor-DEGs (Fig. 2B) were found. Consistently, when DEGs selected by − log_10_(*FDR*) > 2 threshold were indicated in the volcano plot, the number of NAT-DEGs (blue genes one the left panel) was significantly greater than that of tumor-DEGs (red genes on the right panel) (Fig. 2C). In contrast, when log_2_ fold change (FC) was applied along with *FDR*, (e.g., − log_10_(*FDR*) > 2 and abs(log_2_FC > 2), the number of dots representing DEGs in the volcano plot of the tumors became larger than that of the NATs, indicating that the expression level of each gene within the tumor samples is more heterogeneous than NAT samples. The same conclusions were drawn when only protein-coding NAT-DEGs and protein-coding tumor-DEGs were compared (Additional file 1: Fig. S1A, B), consistently showing that the number of NAT-DEGs was significantly higher than that of tumor-DEGs. The significant (*FDR* < 0.01) coding and non-coding DEGs for both NATs and tumors are provided in Tables S1-2 (Additional file 2: Tables S1–2).

Next, we analyzed the same question with a total of fifty-one genes collected from the five commercially available prognostic test kits (Table 3 of Koncina et al. [39], including OncotypeDX, ColoPrint, ColoGuideEx, ColoGuidePro, and ColoDefender). Briefly, after overlapping the fifty-one genes with our two datasets, NAT- and tumor-derived transcriptomes, respectively, the magnitudes of FCs (the lower panel of Fig. 2D) along with *P*-values (the upper panel of Fig. 2D) underlying differences in gene expressions between RC and nonRC samples were estimated, just in the same way as for estimating NAT-DEGs and tumor-DEGs described in Fig. 2A&B. We found that the thirty-three genes of fifty-one genes were more significantly differentially expressed between RC and nonRC samples when using the NAT- than when using the tumor-derived transcriptome (Fig. 2D), consistently the *P*-values significantly deviated from random expectations for both transcriptome datasets but NATs had higher magnitudes than tumors (Fig. 2E). In particular, nine of the fifty-one genes had significantly lower *P*-values and higher FCs in NAT-derived transcriptome while only four genes had significantly lower *P*-values and higher FCs in tumor-derived transcriptome. The lowest five *P*-values were observed for CXCL9, CXCL10, PIM3, DMBT1 and MMP3 in the NAT-derived transcriptomes (Fig. 2D).

### NAT- and tumor-DEGs reveal distinct functions

**Fig. 3:**
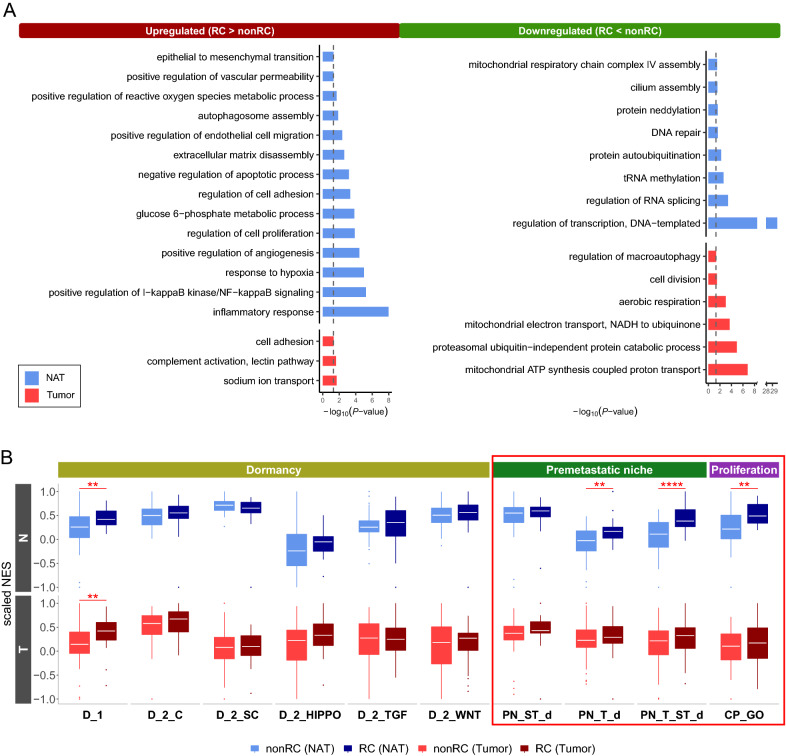
Comparison of statistical characteristics of NAT- and tumor-DEGs. **A** Bar plot of the GO functional terms of NAT-DEGs and tumor-DEGs. Black-dashed lines represent *P* = 0.05. **B** Box plot of the result of single-sample GSEA (ssGSEA) of ‘dormancy’ signatures, ‘premetastatic niche’ signatures, and ‘proliferation’ signatures. The significance of the difference between RC and nonRC states was measured by Wilcoxon rank sum test. **P < 0.01, ***P < 0.001, ****P < 0.0001. Refer to the Methods section for where each signature comes from. D_1, dormancy-associated genes from dormancy study_1; D_2_C, classical dormancy-associated genes from dormancy study_2; D_2_SC, dormancy-related genes revealed by the single cell analysis from dormancy study_2; D_2_HIPPO, Hippo pathway genes from dormancy study_2; D_2_TGF, TGF-beta pathway from dormancy study_2; D_2_WNT, WNT signaling pathway from dormancy study_2; PN_ST_d, stroma-derived ‘premetastatic niche’-associated genes; PN_T_d, tumor-derived ‘premetastatic niche’-associated genes; PN_ST_T_d, stroma- and tumor-derived ‘premetastatic niche’-associated genes; CP_GO, ‘cell proliferation’-associated genes from GO terms

We performed gene ontology (GO) analysis on the NAT-DEGs and the tumor-DEGs, respectively, to investigate how these two types of DEGs differ with respect to gene functions. As a result, interestingly, the functional terms related to tumorigenesis were enriched in both NAT-DEGs and tumor-DEGs, whereas the terms such as inflammatory response, response to hypoxia, and angiogenesis were enriched only in NAT-DEGs (Fig. 3A). Furthermore, when gene expressions were compared between nonRC and RC states, we found that NATs tend to express various kinds of signature genes for ‘premetastatic niche’ and ‘proliferation’ at significantly different levels, whereas no significant differences in tumor tissues (genes in the red box of Fig. 3B). No significant difference exhibited in both NATs and tumor tissues in signature genes for ‘dormancy’ except only one class called ‘D_1’ representing dormancy-associated genes from the ‘dormancy study_1’ signature (see “Methods”) (Fig. 3B).

### NAT-based predictive model effectively predicted the survival of COAD patients when applied to tumor-derived transcriptome data from TCGA

Considering that only the tumor-derived transcriptomes are available in most cases in the clinical setting, we assessed to which our NAT- and tumor-derived models are prognostic in an independent cohort where only tumor-derived transcriptomes are available. For this purpose, we attempted to validate the two prognostic models using the tumor-derived transcriptome data of 450 TCGA-COAD patients as an independent test set.

**Fig. 4:**
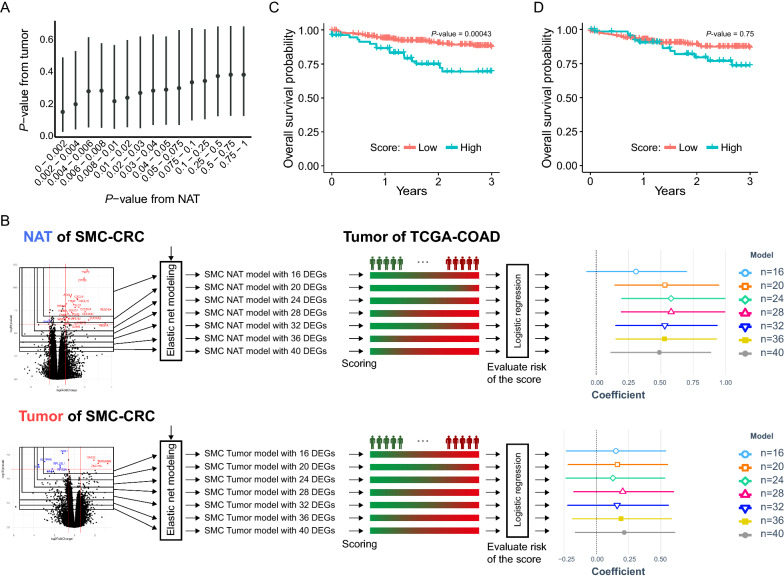
Comparison of prognostic accuracy of NAT- and tumor-based models. **A** The distributions of *P-*values from estimating the NAT-DEGs and *P*-values from estimating the tumor-DEGs are compared. The dot in each bar graph shows the median of *P-*values generated from each tumor-DEG against the *P*-values estimated from NAT-DEGs in the indicated ranges. **B** Schematic of the procedure to test model accuracy of two types of prognostic models, i.e., NAT- and tumor-based model from SMC-CRC (left most panel), applying logistic regression to tumor-derived transcriptomes of TCGA-COAD (middle panel). Note that only 186 tumor-derived transcriptomes harboring information on the patients’ prognosis were used for the logistic regression analysis out of the total 450 TCGA-COAD samples with count information. The beta coefficient and 95% CI of each model are depicted as different point shapes and segments depending on the number of genes used in the model, on the rightmost panel. **C**, **D** Kaplan–Meier plot of survivals of the TCGA-COAD patient groups: the patients with high risk scores (turquoise) and the patients with low risk scores (crimson red) were classified based on the risk scores estimated from (**C**) NAT-based models or **D** tumor-based models

We first investigated how concordant NAT-DEGs and tumor-DEGs were in terms of *P-*values that underlie the estimation of each type of DEGs, and found that they are highly concordant each other (Fig. 4A). We then constructed two elastic net-based machine learning models to predict the recurrence state of CRC, (i) NAT-based elastic net models and (ii) tumor-based elastic net models. We chose the elastic net algorithm, one of regularization-based machine learning algorithms, to build the prognostic models, because it has been reported to outperforms in general other machine learning algorithms such as random forest, support vector machine, and LASSO, etc., when the number of features is much larger than the number of samples [40–44]. For each type, several elastic net models were built using different numbers of DEGs, 16, 20, 24, 28, 32, 36, and 40 DEGs. (Fig. 4B). The recurrence risk score was then calculated for each TCGA sample as the Cosine product of the gene coefficients in the elastic net model and the gene expression in the sample. Finally, a multivariate logistic regression analysis was performed with these estimated risk scores using TNM stage and sex information as covariates in predicting three-year survival of TCGA-COAD patients. Notably, the multivariate logistic regression models built with the risk scores derived from NAT-derived elastic net models produced higher coefficients (~ 0.6) (the upper right panel of Fig. 4B) than those built with the risk scores derived from tumor-derived elastic net models (~ 0.25) (the lower right panel of Fig. 4B). In addition, coefficients of the predicted risk scores generated from all different NAT-derived elastic net models built with different numbers of NAT-DEGs had 95% confidence intervals (CIs) above zero, whereas coefficients generated from all different tumor-derived elastic net models built with different numbers of tumor-DEGs all included zero, without any exception (the far-right panel in Fig. 4B). The NAT-based elastic net model built with 28 DEGs was found to be the best prognostic model in this analysis, and these 28 DEGs contained several genes involved in chemokine activity or insulin-like growth factor receptor binding including NRSN2, CXCL10, CXCL9, NOS2, and TYMP.

We further validated the fact that the NAT-based models outperformed the tumor-based models, even after correcting the batch effect in the two datasets, i.e., the SMC-CRC transcriptomes (i.e., the transcriptomes generated in the present study) and the TCGA-COAD transcriptomes, by testing which one of these two types of models better predicted the three-year survivals of the TCGA-COAD patients, when NAT- or tumor-derived transcriptomes of TCGA-COAD were used as inputs to the models. As shown in Additional file 1: Fig. S2, the NAT-based models performed very well for the NAT-derived transcriptomes of TCGA-COAD as well as tumor-derived transcriptomes of TCGA-COAD, whereas tumor-based model failed to predict the three-year survival even when tumor-derived transcriptomes of TCGA-COAD were used as input.

We also investigated how well the risk scores estimated by the NAT- and tumor-based elastic net models are concordant with the three-year survivals of TCGA-COAD patients. For this purpose, Kaplan–Meier plot analysis was performed after the TCGA-COAD patients were divided into two groups based on the risk scores: the top 20% (i.e., patients with high risk scores) and the remaining 80% (i.e., patients with low risk scores). Interestingly, a significantly different survival between the patients with high risk scores and the patients with low risk scores was observed, only when the risk scores were estimated by NAT-derived elastic net model; when the NAT-based models were used, patients with high risk scores were found to have poor survival rates within 2–3 years (*P* = 0.00043) (Fig. 4C), whereas when tumor-based model used, the difference in survival rates between patients with high and low scores was not statistically significant (Fig. 4D).

Taken together, NAT-derived elastic net models performed better in predicting the three-year survival of TCGA-COAD patients than tumor-derived elastic net models, even when the NAT-derived elastic net models were applied to estimate risk scores using the tumor-derived transcriptomes of TCGA-COAD.

### Infiltrating immune cells are better predictor in NATs than in tumors

**Fig. 5:**
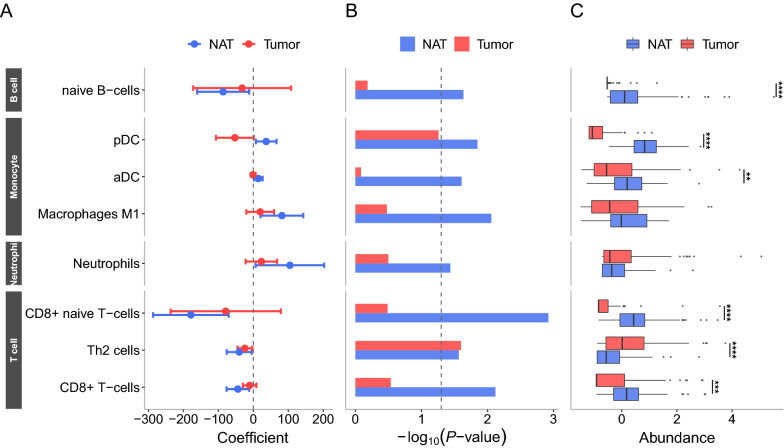
Comparison of immune cell compositions with respect to their prognostic predictability. From each of the NAT- and tumor-driven transcriptomes, the proportions of 29 deconvoluted immune cell types were estimated using xCell. Logistic regression analysis was then applied NATs and tumors to determine whether immune cell composition can differentiate patient recurrence. Eight out of the 29 cell types that were significant at *P* < 0.05 in either NATs or tumors are shown. **A** The beta coefficient and 95% CI and **B** the *P-*value of each cell type are depicted. **C** Z-score normalized cell proportions per cell type are depicted, and the difference of cell proportions between NAT and tumor samples was tested by the paired-sample t-test. **P < 0.01, ***P < 0.001, ****P < 0.0001. pDC, plasmacytoid dendritic cells; aDC, activated dendritic cells; Th2 cells, T helper 2 cells

Based on the finding that the compositions of tumor-infiltrating immune cells, such as T cells, B cells, and macrophages are associated with patient survival in several cancer types including CRC [39, 45–49], we asked whether compositions of tumor-infiltrating immune cells in NATs could also provide useful information for predicting prognosis. To address this, using xCell—a deconvolution tool [50] we first inferred cell type composition in the NATs and tumor tissues of the 73 CRC patients. A total of 29 immune cell types were identified in NATs and tumors (Additional file 2: Table S3). After the proportion of each immune cell type was estimated for each patient, logistic regression analysis was performed to determine how well the immune cell proportions in NATs or in tumors can distinguish RC and nonRC states, with TNM stage and sex used as covariates. As a result, four out of the 29 immune cell types (naïve CD8 + T cells, CD8 + T cells, and Th2 cells, and naïve B cells), and three other cell types including macrophage and dendritic cells (M1 macrophages, aDCs and pDCs), and neutrophils were found significant predictors with respect to beta coefficients of recurrence (Fig. 5A and Additional file 2: Table S4), at least for one of the NATs and tumors. More importantly, seven types of cells had greater significance in NATs (i.e., lower *P-*values) than in tumors to discriminate between RC and nonRC conditions (Fig. 5B). Consistently, the proportions of these cell types were higher in NATs than in tumors (Fig. 5C). All these results strongly indicate that compositions in tumor-infiltrating immune cell of NATs could also provide information regarding the prognosis of patients.

### Examination of the efficacy of NAT- and tumor-derived transcriptomes in other TCGA cancers

Next, we examined whether the finding based on the SMC-CRC-derived transcriptomes generalize to other cancer types. For this purpose, NAT- and tumor-derived transcriptomes were downloaded from other cancer types including lung cancers (LUAD), breast cancers (BRCA), and liver cancers (LIHC) in the TCGA database. One caveat in exploring this question is that in TCGA, NAT-derived transcriptomes are severely lacking compared to tumor-derived transcriptomes in most cancer types. As shown in Table S5 (Additional file 2: Table S5), while 41 COAD, 58 LUAD, 99 BRCA, and 50 LIHC NAT-derived transcriptome data were available, only 22 COAD, 44 LUAD, 74 BRCA, 41 LIHC paired samples had survival information. The numbers of samples with death events were 8, 18, 12, and 23 in each cancer. Due to the small number of NAT data for the TCGA cancers, instead of building the elastic net-based machine learning models, we chose to perform Cox regression analysis to validate our conclusion. Even in Cox regression, only four cancer types including COAD, LUAD, BRCA, and LIHC were subjected to the analysis, as the previous study showed that Cox regression requires at least 5 to 10 events per variable [51].

**Fig. 6:**
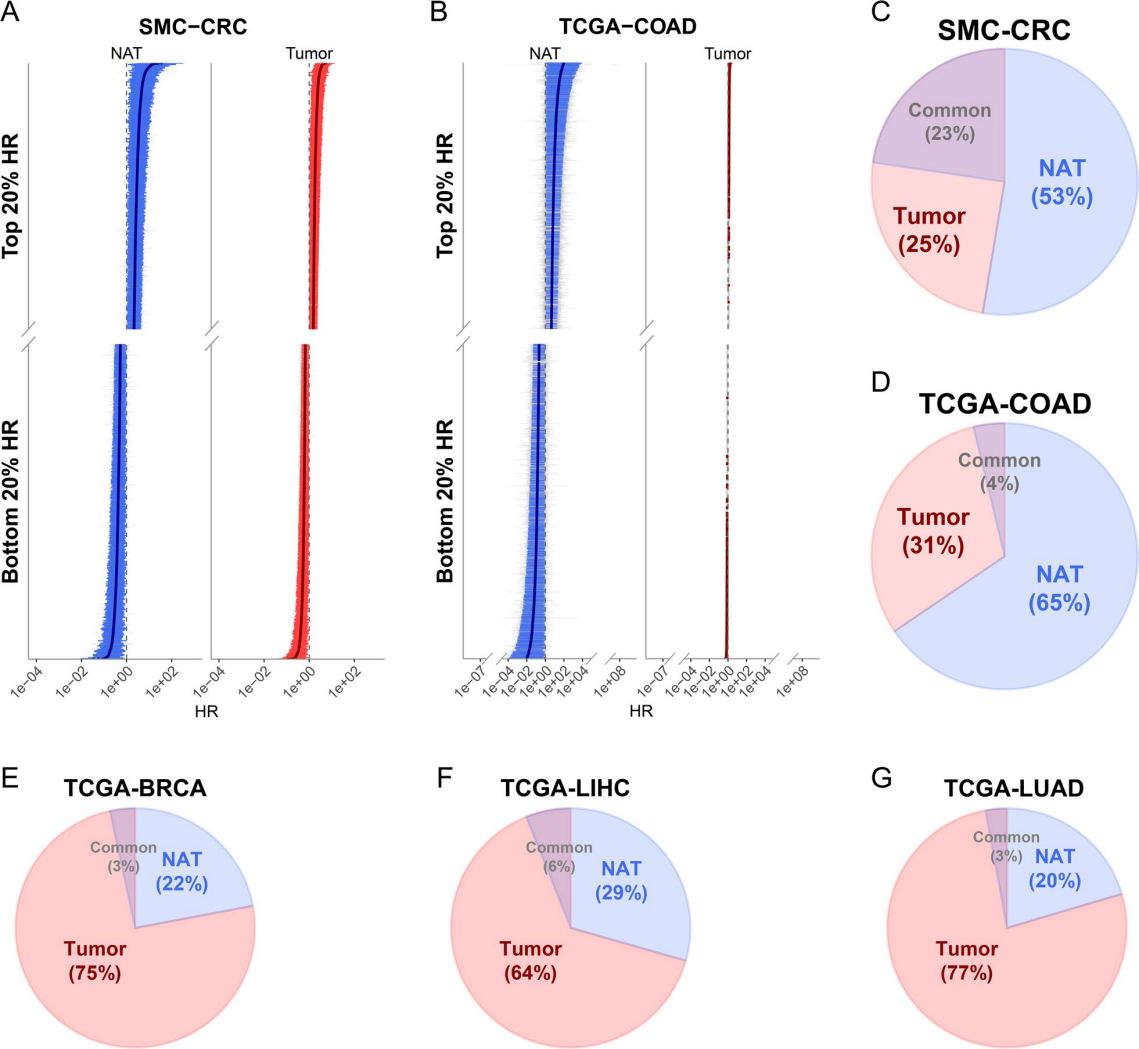
Comparison of proportions of survival-associated genes between NATs and tumors in different TCGA cancer types. **A** HR and the 95% CI of each gene from SMC-CRC samples are depicted. Only genes with top and bottom 20% HR in NATs (left panel) and tumors (right panel) are shown, respectively. Significant survival-associated genes (*P* < 0.05; Cox regression analysis) are colored blue for NAT and red for tumor. **B** Same as (**A**) but for TCGA-COAD samples. Note that 79 outlier genes with HR > 100 or HR < 0.01 in either NATs or tumors were removed. **C–G** Pie charts for the significant survival-associated genes (*P-*value < 0.05; Cox regression analysis) in (**C**) SMC-CRC, **D** TCGA-COAD, **E** TCGA-BRCA, **F** TCGA-LIHC, and **G** TCGA-LUAD are shown

For these four cancer types in the TCGA, we examined whether NAT- or tumor-derived transcriptomes in each cancer type has greater proportions of genes that were significantly associated with survival. For this purpose, we first estimated hazard ratio (HR) of each gene through Cox regression analysis using age, TNM stage, and sex information as covariates, respectively, from NAT- and tumor-derived transcriptome. Subsequently, we compared the proportions of genes with significant HRs between NAT and tumor in each cancer type. Note that SMC-CRC and TCGA-COAD were included as a kind of positive control in this HR test. Encouragingly, we found that the proportions of genes with significant HRs in both SMC-derived CRC (Fig. 6A) and TCGA-COAD (Fig. 6B) were significantly higher in NAT- than in tumor-derived transcriptomes; 53% NAT vs. 25% tumor for SMC-CRC samples (Fig. 6C), and 65% NAT vs. 31% tumor for TCGA-COAD samples (Fig. 6D). Even if TCGA-COAD tumor samples were adjusted by tumor purity data [52], the result was the same as above (Additional file 1: Fig. S3). However, LUAD, BRCA, and LIHC exhibited completely opposite to SMC-CRC- or TCGA-COAD-based finding, so the proportion of genes with significant HRs was significantly higher in tumor- than NAT-derived transcriptome; 22% NAT vs. 75% tumor for BRCA, 29% NAT vs. 64% tumor for LIHC, and 20% NAT vs. 77% tumor for LUAD (Fig. 6E–G). These results suggest that a greater clinical information in the NAT compared to tumor transcriptome may be true only in some of the cancers, specifically, CRC in this analysis.

## Discussion

Discovering prognostic factors or developing prognostic machine learning models has recently been a major focus in cancer studies [53–55]. However, as with many other cancers, numerous prognosis prediction methods of CRC, such as tumor locations, types of genetic mutations, degree of chromosomal instability, were largely inconsistent [56]. For instance, some studies have shown a better prognosis for left-sided tumors than right-sided ones [57], while other studies have shown the opposite [58]. Changes in immune or inflammatory cell composition in the tumor microenvironment have also been controversial in related to the prognosis of patients with CRC [48, 59–62]. We noticed that various transcriptomes, epigenomes, and cell composition data used as source materials in previous studies to develop prognostic models and biomarkers were primarily derived from tumors [63–65]. Therefore, we suspected that part of the inconsistencies in various prognostic methods may be due to high inter- and intra-tumor heterogeneity.

In the present work, we showed that tumor tissues have higher expressional variances than NAT samples, by obtaining NAT-DEGs and tumor-DEGs under two types of thresholds with a different strength of stringency, (i) *FDR* < 0.01 only, and (ii) both *FDR* < 0.01 and log_2_FC > 2, were applied (Fig. 2C). A significantly larger number of DEGs was generated from the NAT-derived transcriptomes than from the tumor-derived transcriptomes when the less stringent threshold was applied, but vice versa when more stringent thresholds were used. This result indicates that a small number of tumor samples experienced large-scale changes in gene expression between the RC and nonRC states, resulting in higher FC values on average, but the size of changes was not even across all the tumor samples, which caused to failure in discriminating the recurrence states. On the contrary, NATs showed expression changes between different recurrence states at small sizes but significant changes with relatively uniform values across all samples, and consequently, NAT-derived transcriptomes turned out to become more informative in building the prognostic models.

NAT is a histologically normal tissue, but it has been reported that NAT has molecular characteristics that distinguish it from healthy normal tissues. Aran et al.’s conclusion that NAT is an intermediate state between tumor and healthy normal states agreed with the concept of field cancerization, which was reviewed by Curtius et al. [66]. Field cancerization argues that tumor formation begins long before clinically detectable morphological changes occur, as observed in several cancers, including colon, lung, and prostate cancers [66]. It seems that NAT is the tissue experiencing field cancerization, in which stepwise molecular changes accumulate during tumor formation through the process of cancer evolution [67, 68]. It is expected that the stepwise field cancerization process varies in the NAT of each patient because the tumor of each patient develops as a result of different initial mutations and various subsequent evolutionary paths that are influenced by patient-specific natural selection. The results of the present work show that the gene expression perturbations that occur in NAT have more prognostic value than those in tumor tissue and in good agreement with the concept of field cancerization. In other words, NAT in a CRC patient without recurrence appears to have specific types of gene expression changes that drive tumor growth, and similarly, NAT in a CRC patient with recurrence seem to have other specific types of gene expression changes driving tumor growth. This suggests that prognosis can be predicted based on gene expression profiles of NAT from surgically resected samples during therapeutic resection of early-stage CRC, long before tumor recurrence and metastasis. Consistently, Facista et al. [69] showed that early-stage colon cancer resection samples had a region (or NAT) with abnormally reduced expression of DNA repair enzyme genes (ERCC1, Pms2, and Xpf) when a series of approximately 15 sequential tissue sections (4 microns for each section) were examined. Our analysis focused on NAT-derived transcriptomes makes sense in that respect.

It is noteworthy that we validated the performance of the elastic net-based prognostic models using the TCGA-COAD dataset with survival information because no independent datasets with recurrence information were available from TCGA or other curated cancer databases. While survival period is strongly correlated with recurrence status, it may not always be consistent with the RC/nonRC states. Nonetheless, the NAT-based elastic net models we built predicted well the survivals of patients, such that the patients in a high risk group had significantly worse survivals than the patients with a low risk group (Fig. 4). An interesting point in this validation analysis was that we validated the NAT-based elastic net model against the 450 tumor-derived transcriptomes data rather than NAT-derived transcriptome data because of the sample accessibility in general clinical settings. In Fig. 6, we showed that, in both SMC-CRC and TCGA-COAD datasets, NAT-derived transcriptomes are more informative in differentiating CRC prognosis than tumor-derived transcriptome, by showing that the proportion of genes with significant HR estimated using Cox regression is significantly greater in NAT-derived transcriptome than in tumor-derived transcriptome.

Unexpectedly, three other cancer types including LUAD, BRCA, and LIHC, i.e., cancer types in TCGA with relatively large numbers of NATs, did not reiterate the conclusion made by CRC dataset in the present work (Fig. 6E–G). In other words, tumor-derived transcriptomes rather than NAT-derived transcriptomes have significantly more genes with significant HR in these three types of cancers. One possible cause for this observation could be that the numbers of genes that have alterations in gene expressions or the sizes of the alterations in the NATs of these cancers may be smaller than the number or the size of alterations in gene expression in the NAT of CRC. Given that previous studies in breast cancers and liver cancers have already shown that gene expressions in NATs harbor information reflecting the cancer prognosis [5, 37, 70], it should not be wrong to expect that the scenario based on CRC is applicable to other cancer types. A possible cause for the inconsistency would be related to the distance of the NATs from the edge of the resected tumor, which varies across cancer types. The NATs from relatively close to the tumor tissue are expected to show greater alterations in gene expressions. Another possible cause may be related to the extent to which each cancer type harbor alterations in gene expressions, as suggested by Aran et al. [1] that NATs in different cancer types tend to carry different degrees of alterations in gene expression, and the NATs of CRC harbor more shifted transcriptomic profiles toward tumor than other cancers.

## Conclusions

Our study investigated, for the first time, the value of NAT-derived transcriptomes compared to tumor-derived transcriptome in predicting clinical prognosis for CRC patients. By building elastic net-based machine-learning models, we found that NAT-based models outperformed tumor tissue-based models in predicting the recurrence states of CRC patients. We validated our models using independent transcriptome data from the TCGA-COAD cohort, showing that NAT-based model effectively predicted the survival of CRC patients, even when applied to tumor-derived transcriptomes. Additionally, Cox regression analysis confirmed that the proportion of genes with significant hazard ratios was higher in NATs than in tumors in CRC. While the NATs are believed to harbor a tumor-supportive microenvironment, in transcriptome-based studies thus far, NATs are often used as a proxy for non-malignant or healthy control. Our study clearly challenges this assumption and highlights the importance of using NAT-derived transcriptome data for understanding oncogenesis, tumor aggressiveness, and therapy response in CRC.

## Methods

### Tissue samples

This study was performed in accordance with the principles of the Declaration of Helsinki and was approved by the ethics committee of SMC in South Korea (No. SMC-2018-04-074-004). A total of 160 tissue samples collected from 80 Korean CRC patients (Table 1) were retrieved from the biobank at SMC. Tumor tissue samples and matched normal tissues had been originally collected from patients who signed a consent form for the donation of specimens for research purposes and underwent surgery for CRC at SMC from Jan 2011 to Dec 2013. Patients diagnosed with stage I-III colorectal cancer and monitored for recurrence at least three years were included. Among the 80 patients, 48 were retrospectively characterized as CRC with nonrecurrence states and 25 as CRC with recurrence states based on the recurrence of during the follow-up period. The tissue samples at the SMC biobank were collected and stored in the vapor phase of liquid nitrogen.

### RNA sequencing

Total RNA was then isolated using an AllPrep DNA/RNA kit (Qiagen, Santa Clarita, CA, USA) according to the manufacturer’s protocol. To access the quality of RNA, RNA integrity number (RIN) was measured using a 2200 TapeStation Instrument (Agilent Technologies, Santa Clara, CA, USA). Samples with an RNA integrity number (RIN) less than seven were excluded from subsequent library preparation. We created libraries using a TruSeq kit (Illumina, San Diego, CA, USA). For each sample, 500 ng of total RNA was used to generate libraries with different indexing adaptors in one sequencing run according to the manufacturer’s protocol. The library was purified with AMPure beads and quantified using a Qubit 2.0 Fluorometer with a dsDNA HS Assay Kit (Thermo Fisher Scientific, Waltham, MA, USA). The size distribution was analyzed using a 2200 TapeStation Instrument (Agilent Technologies). Based on DNA concentration and average fragment size, libraries were pooled and denatured as previously described [71]. The libraries were sequenced on a HiSeq 2500 system using 100-bp paired-end sequencing (Illumina) to generate approximately 50–80 million reads per sample.

### Quantifying mRNA and identifying DEGs

The STAR [72] and HTSeq [73] bioinformatics pipelines, respectively, were used to map and count the raw reads. In brief, quality control of the raw reads was performed with the FastQC program (https://www.bioinformatics.babraham.ac.uk/) [74]. Subsequently, Trimmomatic (v0.38) [75] was applied to remove contaminating adaptor sequences and unpaired reads. The ‘genomeGenerated’ option of STAR v020201 was then used to index the reference genome, GRCh38.p12. After the clean reads in the FASTQ file were aligned to the index genome, ‘htseq-count’ was used to count the aligned reads. To annotate the names of mRNAs, the GTF file of Ensembl (GENCODE v29) was used. We removed genes with zero counts in more than 70% of samples. The DESeq2 [76] R package was used to identify DEGs after the read counts were normalized.

### ssGSEA of ‘dormancy’, ‘premetastatic niche’, and ‘proliferation’ signatures.

Each signature was collected from the literature search on PubMed and named it as following; ‘D_1’ (i.e., dormancy study_1) signature was retrieved from [75]. All signatures prefixed with ‘D_2’ (i.e., dormancy study_2), i.e., signature genes that are all related to dormancy, were defined and collected by the single cell analysis of [77]: ‘D_2_C’, a classical dormancy signature that contains well-known dormancy-associated genes; ‘D_2_SC’, dormancy-related genes revealed by the single cell analysis; ‘D_2_HIPPO’, ‘D_2_TGF’, and ‘D_2_WNT’, genes associated with the Hippo pathway, TGF-beta pathway, and WNT signaling pathway, respectively. All ‘premetastatic niche (prefixed with PN)’ signatures were from the review of Liu and Cao (2016) [78]: ‘PN_ST_d’ and ‘PN_T_d’, ‘premetastatic niche’-associated genes defined from the studies using stroma and tumor samples, respectively; ‘PN_T_ST_d’, genes associated with premetastatic niche in both tumor and stroma samples. Finally, ‘CP_GO’, signature genes were obtained from ‘CELL_PROLIFERATION_GO_0008283’ (http://amigo.geneontology.org/amigo/term/GO:0008283) of the Molecular Signatures Database (MsigDB) [79–81]. ssGSEA was then performed by the ‘gsva’ function of GSVA R package [82] for these signature genes in NAT- and tumor-derived transcriptomes. Enrichment scores (ES) of all sample are z-scores normalized per each signature, resulting in normalized ES (NES).

### Processing of TCGA RNA-seq data

After we obtained the raw count data of tumor samples and matched normal samples of six different cancer types (COAD, LUAD, BRCA, LIHC, PRAD (i.e., prostate cancers), and THCA (i.e., thyroid cancers)) from the TCGA repository (https://portal.gdc.cancer.gov/), the transcriptomes from four cancer types including COAD, LUAD, BRCA, and LIHC were used for further analyses after two cancer types lacking the numbers of NATs, PRAD and THCA, were excluded. Genes with zero counts in more than 70% of samples were removed. To ensure that the sample normalization was applied to all samples, both the gene expression data of the tumor and NAT samples of the SMC cohort and the TCGA cohort were placed in one basket and then read count values were normalized using DESeq2. To eliminate the sequencing center-originated gene expression difference, the transcriptome data were again divided into the original cohorts and tissue groups and then standardized at the gene level.

### Construction of prediction models and evaluation of model accuracy

The elastic net algorithm, an algorithm that is particularly useful when predictor variables outnumber the samples because it regularizes the model by giving an appropriate penalty to large coefficients, was chosen to construct prognostic machine learning models using NAT-DEGs and tumor-DEGs. The type and degree of penalty were adjusted by the cross-validation method. The alpha value to adjust the balance between the L1 and L2 norms was tested from 0.1 to 1.0 in increments of 0.1. For each alpha value, the lambda value that minimized the misclassification error was determined. Predictor variables were selected from the identified DEG sets. Seven different elastic net-based models were built for each of the NAT and tumor conditions (i.e., 14 models in total) using different numbers of DEGs (n = 16, 20, 24, 28, 32, 36, and 40), which were chosen based on statistical significance. Then, each of the 14 predictive models was used to analyze the 450 TCGA-COAD samples to estimate the recurrence risk scores for each individual. Subsequently, the recurrence risk score was subjected to logistic regression to test its association with survival time in the TCGA-COAD dataset; in this analysis, the patients with data in the TCGA-COAD dataset were dichotomized into good and poor prognosis groups as the response variable. Patients censored within three years were excluded.

### Statistical analysis

All statistical analyses were performed using the R programming language (version 4.0.3) [83]. Various plots were constructed using the ‘ggplot2’ R package (version 3.3.3) [84]. GO analysis was done using the Database for Annotation, Visualization, and Integrated Discovery (DAVID) tool [85]. Elastic net analysis was performed using the ‘glmnet’ R package (version 4.1) [86], and logistic regression analysis were performed using the ‘stats’ R Base package. The forward selection for logistic regression model and the calculation of AIC values were done by ‘MASS’ R package (version 7.3.55) [87]. The batch effect between SMC cohort and TCGA cohort was corrected by the ‘sva’ R package (version 3.40.0) [88]. HR was estimated from the survival analysis by Cox-regression using the ‘survival’ R package (version 3.3.1) [89, 90]. Kaplan–Meier plot was drawn using the ‘survminer’ R package (version 0.4.9) [91].

## Supplementary Information

**Additional file 1: Figure S1** Comparison of *P*-values and *FDR*s between NAT-DEGs and tumor-DEGs. The same way of analysis that was done for Fig. 2, but using only protein-coding genes. Refer to the legends of Fig. 2. **Figure S2** The accuracy of NAT- and tumor-based model after correcting the batch effect. The beta coefficients and 95% CIs are estimated, respectively, (A) when SMC NAT-based model was tested on the NAT-derived transcriptome of TCGA-COAD, (B) when SMC NAT-based model was tested on the tumor-derived transcriptome of TCGA-COAD, and (C) SMC tumor-based model was tested on the tumor-derived transcriptome of TCGA-COAD. **Figure S3** Comparison of proportions of genes associated with the survival of patients between NATs and tumors in TCGA-COAD with tumor purity adjusted. The same way of analysis that was done for Fig. 6, but with tumor purity adjusted. Refer to the legends of Fig. 6

**Additional file 2: Table S1** Differential expression analysis result of protein-coding and noncoding genes in NAT-derived transcriptome. **Table S2** Differential expression analysis result of protein-coding and noncoding genes in tumor-derived transcriptome. **Table S3** Cell type proportion predicted by xCell. **Table S4** Summary of logistic regression results with cell type proportion as an independent variable and prognosis as dependent variable. **Table S5** The numbers of samples in six different cancer types of TCGA cohorts.

## Data Availability

The dataset supporting the conclusions of this article is available in the European Genome-Phenome Archive (https://ega-archive.org/) repository, https://ega-archive.org/datasets/EGAD00001006985.

## Abbreviations

BRCA: Breast invasive carcinoma
CI: Confidence interval
COAD: Colorectal adenocarcinoma
CRC: Colorectal cancer
DEG: Differentially expressed gene
FC: Fold change
GO: Gene ontology
HR: Hazard ratio
LIHC: Liver hepatocellular carcinoma
LUAD: Lung adenocarcinoma
NAT: Normal tissues adjacent to tumors
nonRC: Nonrecurrent
RC: Recurrent
SMC: Samsung Medical Center
TCGA: The Cancer Genome Atlas
