# Correction: Transcriptomes of the tumor-adjacent normal tissues are more informative than tumors in predicting recurrence in colorectal cancer patients

**DOI:** 10.1186/s12967-023-04124-4

**Published:** 2023-05-05

**Authors:** Jinho Kim, Hyunjung Kim, Min‑Seok Lee, Heetak Lee, Yeon Jeong Kim, Woo Yong Lee, Seong Hyeon Yun, Hee Cheol Kim, Hye Kyung Hong, Sridhar Hannenhalli, Yong Beom Cho, Donghyun Park, Sun Shim Choi

**Affiliations:** 1 Precision Medicine Center, Future Innovation Research Division, Seoul National University Bundang Hospital, Seongnam, 13620 Korea; 2 Division of Biomedical Convergence, College of Biomedical Science, Institute of Bioscience & Biotechnology, Kangwon National University, Chuncheon, 24341 Korea; 3 Center for Genome Engineering, Institute for Basic Science, 55, Expo‑ro, Yuseng‑gu, Daejeon, 34126 Korea; 4 Samsung Medical Center, Samsung Genome Institute, Seoul, 06351 Korea; 5 Department of Surgery, Samsung Medical Center, Sungkyunkwan University School of Medicine, Seoul, 06351 Korea; 6 Samsung Medical Center, Institute for Future Medicine, Seoul, 06351 Korea; 7 Cancer Data Science Lab, Center for Cancer Research, National Cancer Institute, Bethesda, MD 20814 USA; 8 Department of Health Sciences and Technology, SAIHST, Sungkyunkwan University, Seoul, 06351 Korea; 9 Geninus Inc, Seoul, 05836 Korea

**Correction: Journal of Translational Medicine (2023) 21:209** 10.1186/s12967-023-04053-2

Following publication of the original article [1], we have been notified that the affiliations of some authors were incorrectly listed. The correct affiliations should be:

^1^Precision Medicine Center, Future Innovation Research Division, Seoul National University Bundang Hospital, Seongnam 13620, Korea: Jinho Kim^†1^, Heetak Lee^1,3^.

^2^Division of Biomedical Convergence, College of Biomedical Science, Institute of Bioscience & Biotechnology, Kangwon National University, Chuncheon 24341, Korea: Hyunjung Kim^†2^, Min-Seok Lee^2^, Sun Shim Choi^2*^.
