# Supplementary material for: Transcriptomes of the tumor-adjacent normal tissues are more informative than tumors in predicting recurrence in colorectal cancer patients
Source: J Transl Med. 2023 Mar 21;21:209. doi: 10.1186/s12967-023-04053-2 (PMC10029176; doi:10.1186/s12967-023-04053-2)
Supplement: Supplementary file 1 — Additional file 1: Figure S1 Comparison of P-values and FDRs between NAT-DEGs and tumor-DEGs. The same way of analysis that was done for Fig. 2, but using only protein-coding genes. Refer to the legends of Fig. 2. Figure S2 The accuracy of NAT- and tumor-based model after correcting the batch effect. The beta coefficients and 95% CIs are estimated, respectively, (A) when SMC NAT-based model was tested on the NAT-derived transcriptome of TCGA-COAD, (B) when SMC NAT-based model was tested on the tumor-derived transcriptome of TCGA-COAD, and (C) SMC tumor-based model was tested on the tumor-derived transcriptome of TCGA-COAD. Figure S3 Comparison of proportions of genes associated with the survival of patients between NATs and tumors in TCGA-COAD with tumor purity adjusted. The same way of analysis that was done for Fig. 6, but with tumor purity adjusted. Refer to the legends of Fig. 6 [file 12967_2023_4053_MOESM1_ESM.docx]

**Comparison of recurrence prediction power of machine learning models built with normal and tumor tissue-driven transcriptomes of colorectal cancers**

Jinho Kim^1^, Hyunjung Kim^2^, Min-Seok Lee^2^, Heetak Lee^1,3^, Yeon Jeong Kim^4^, Woo Yong Lee^5^, Seong Hyeon Yun^5^, Hee Cheol Kim^5^, Hye Kyung Hong^6^, Sridhar Hannenhalli^7^ ,Yong Beom Cho^5*^, Donghyun Park^8*^, and Sun Shim Choi^2*^

^1^Precision Medicine Center, Future Innovation Research Division, Seoul National University Bundang Hospital, Seongnam, 13620 Korea

^2^Division of Biomedical Convergence, College of Biomedical Science, Institute of Bioscience & Biotechnology, Kangwon National University, Chuncheon 24341, Korea

^3^Center for genome Engineering, Institute for Basic Science, 55, Expo-ro, Yuseng-gu, Daejeon 34126, Korea

^4^Department of Surgery, Samsung Medical Center, Sungkyunkwan University School of Medicine, Seoul 06351, Korea

^5^Institute for Future Medicine, Samsung Medical Center, Seoul 06351, Korea

^6^Department of Health Sciences and Technology, SAIHST, Sungkyunkwan University, Seoul 06351, Korea

^7^Cancer Data Science Lab, Center for cancer research, National Cancer Institute, Bethesda, MD 20814

^8^Geninus Inc., 05836 Korea

*Corresponding authors


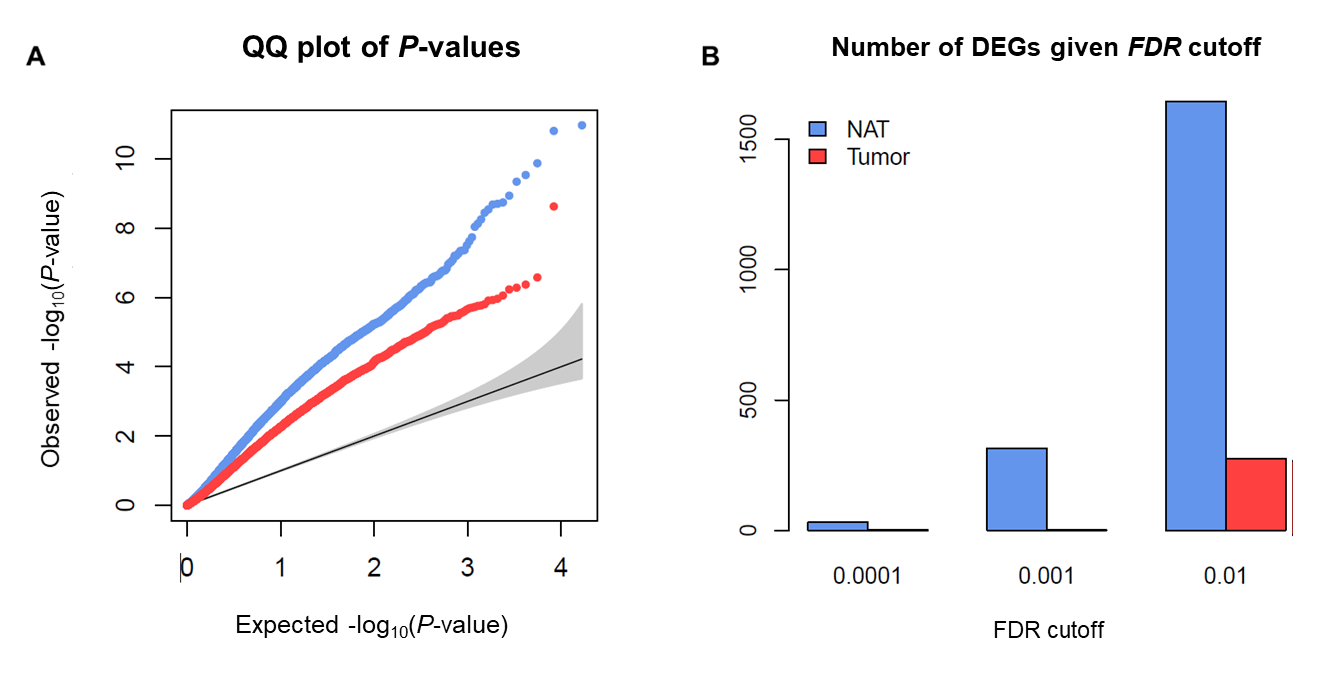


**Figure S1. Comparison of *P*-values and *FDR*s between NAT-DEGs and tumor-DEGs**

The same way of analysis that was done for Figure 2, but using only protein-coding genes. Refer to the legends of Figure 2.

**
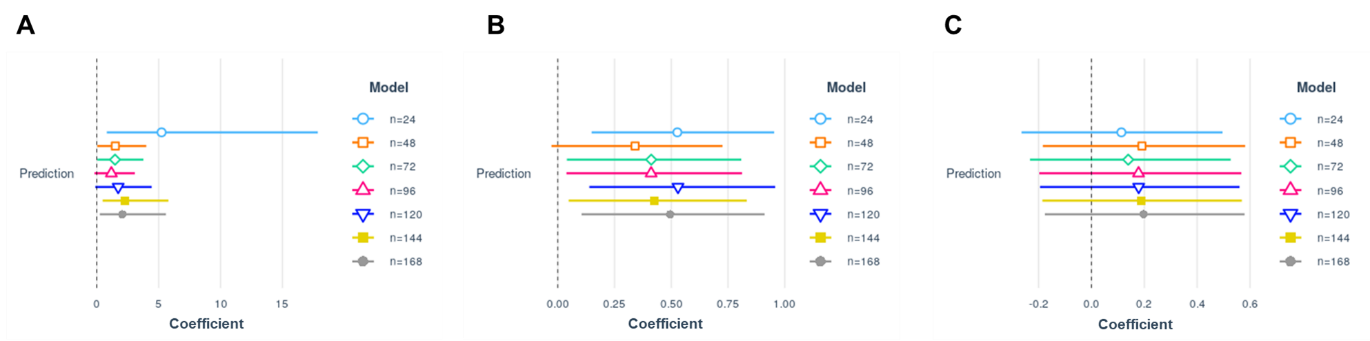
**

**Figure S2. The accuracy of NAT- and tumor-based model after correcting the batch effect**

The beta coefficients and 95% CIs are estimated, respectively, **(A)** when SMC NAT-based model was tested on the NAT-derived transcriptome of TCGA-COAD, **(B)** when SMC NAT-based model was tested on the tumor-derived transcriptome of TCGA-COAD, and **(C)** SMC tumor-based model was tested on the tumor-derived transcriptome of TCGA-COAD.


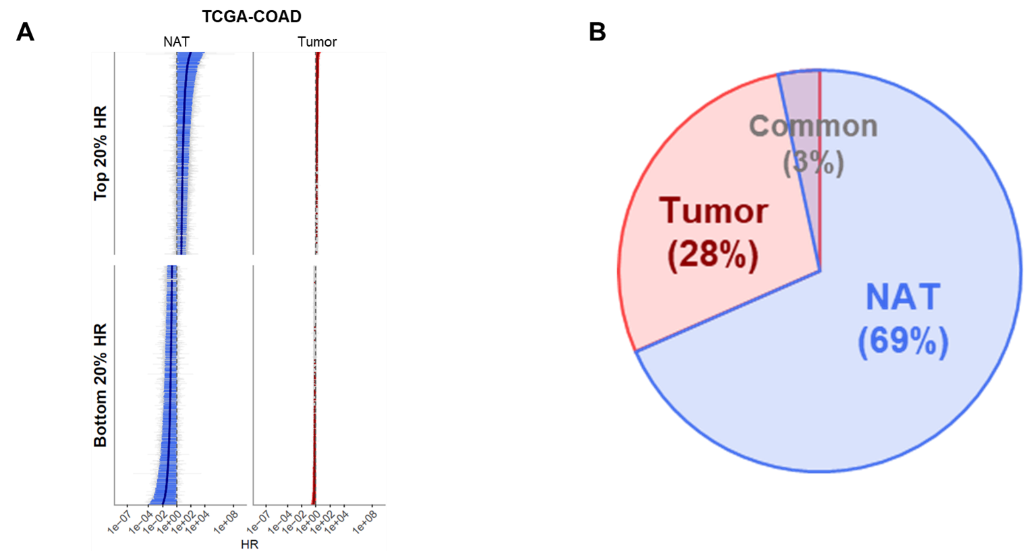


**Figure S3. Comparison of proportions of genes associated with the survival of patients between NATs and tumors in TCGA-COAD with tumor purity adjusted**

The same way of analysis that was done for Figure 6, but with tumor purity adjusted. Refer to the legends of Figure 6.
